# Content and Effect of Introduction Programmes to Increase Retention and Decrease Turnover of Newly Graduated Nurses in Hospitals: Umbrella Review

**DOI:** 10.1111/jocn.17494

**Published:** 2024-10-25

**Authors:** Connie Berthelsen, Carrinna Aviaja Hansen

**Affiliations:** ^1^ Medical Department Zealand University Hospital Koge Køge Denmark; ^2^ Department of Regional Health Research University of Southern Denmark Odense Denmark; ^3^ Department of Orthopaedic Surgery Zealand University Hospital, Køge Køge Denmark

**Keywords:** AMSTAR 2, introduction programmes, mentorship, newly graduated nurses, preceptorship, retention, turnover, umbrella review

## Abstract

**Aim:**

The aim of this study is to combine and compare results from systematic reviews reporting the content and effect of programmes for the introduction of newly graduated nurses employed in hospital settings on increasing retention and decreasing turnover.

**Design:**

An umbrella review.

**Methods:**

The electronic databases of PubMed/MEDLINE, CINAHL and PhycInfo were searched in January 2023 for eligible systematic reviews. The search string consisted of keywords extracted from the PICOS inclusion criteria. The AMSTAR 2 instrument was used for critical appraisal of the methodological quality of the eligible systematic reviews. The process and results of the review were presented using a narrative description of the data.

**Results:**

Five systematic reviews, reporting 84 intervention studies evaluating nine types of introduction programmes from 2001 to 2018, were included in the umbrella review. All nine programme types were executed by nurses in a preceptor or mentor role and the content was directed towards training of the preceptor/mentor and introduction of the newly graduated nurses. The nine programmes showed overall positive effects on retention and turnover.

**Conclusions:**

Mentorship and Preceptorship were the most frequently evaluated programmes in the included intervention studies of the five reviews. However, the lack of transparency of the reviews and the bias of the intervention studies within the reviews, made it difficult to conclude specific effects of the content of the nine programmes.

**Registration:**

The protocol for the umbrella review is registered with Open Science Framework (https://OSF.IO/DXYS4).

**Impact:**

A weak introduction to hospital employment of newly graduated nurses may decrease retention and increase turnover. Structured and personal introduction by a mentor can have an effect on the newly graduated nurses' intentions to stay in their hospital care position.

**Reporting Method:**

AMSTAR 2.

**Patient or Public Contribution:**

None.

**Summary**

- What does this paper contribute to the wider global clinical community?
  ○ Introduction programmes for newly graduated nurses can affect increased retention and decreased turnover but must be combined with knowledge of the local culture and context.
  ○ Personal relationship between the mentor and the newly graduated nurse is imperative for a successful introduction to hospital practice and the social work environment.
  ○ Stronger intervention designs of programmes for introduction are needed to further support the retention of newly graduated nurses.

## 1 Introduction

Nursing shortage in hospitals is a global problem and is related to a negatively skewed turnover (Dewanto and Wardhani 2018; Falatah and Salem 2018). Turnover is defined as the result of the departure of employees and the entry of others to replace them in their role at work, constituting the flow of staff within an organisation (Pedrosa et al. 2021). If more nurses are departing rather than staying, it can have dire consequences for patients, the economy and the productivity of healthcare organisations (White, Aiken, and McHugh 2019). The human consequences are extensive and can be related to poorer patient outcomes such as pain, pressure ulcers, medication errors and patient satisfaction (He et al. 2016) as well as lower patient care quality, poor continuity of care and increased morbidity, which are all related to nursing shortage (Fallatah, Laschinger, and Read 2017).

Newly graduated nurses are often employed in hospital departments without adequate training and preparation for complex patient care (Kenny et al. 2021). Newly graduated nurses describe the process of entering the hospital workplace as difficult and stressful (Phillips, Kenny, and Esterman 2017) because they often find themselves in unfamiliar situations (Kenny et al. 2021). An umbrella review of newly graduated nurses' experiences of providing direct care in hospital settings found that they felt a lack of competencies, emotional distress and in need for support (Kaldal et al. 2023). As a result of their new employment, newly graduated nurses can quickly feel overwhelmed and discouraged due to stress, which can cause them to leave their jobs within the first year or leave the nursing profession for good (Sandler 2018). A supportive transition from student to nurse must therefore be in place to strengthen the newly graduated nurses' introduction to hospital practice and to increase their retention (Hampton, Smeltzer, and Ross 2020).

### 1.1 Content of Introduction Programmes

A large variety of introduction programmes for newly graduated nurses to practice is used in clinical practice. The programmes are used to ensure a safe and thorough introduction and transition to the new workplace for the newly graduated nurses and to strengthen the professional socialisation process that helps new employees fit into the new organisation (Hampton, Smeltzer, and Ross 2020). However, the introduction programmes also focus on more personal aspects of transition to practice by building the newly graduated nurses' confidence (Cadmus, Bohnarczyk, and de Cordova 2023), focusing on social of psychological development and stress management (Melissant et al. 2014).

The literature presents numerous terms used to describe introduction programmes and their content for newly graduated nurses to practice. These include Preceptorship‐ (Edward et al. 2017), Mentorship‐ (Kakyo, Xiao, and Chamberlain 2022), Residency (Eckerson 2018), Internship and Externship programmes (Kenny et al. 2021). The content of the programmes often show similarities according to fixed traines in a preceptorship and/or mentoring role (Edward et al. 2017; Eckerson 2018; Kenny et al. 2021) where preceptors are pre‐selected by the department management and mentors are selected by the newly graduated nurses. Similarities were also found in establishing a relationship between the newly graduated nurse and the trainer (Edward et al. 2017; Kakyo, Xiao, and Chamberlain 2022), theoretical learning, and skills (Eckerson 2018; Kenny et al. 2021) as well as competency development of the newly graduated nurses (Edward et al. 2017; Eckerson 2018; Kenny et al. 2021), and their trainers (Edward et al. 2017).

### 1.2 Effect of Introduction Programmes

Introduction programmes are often evaluated through experimental studies investigating the effect on retention and turnover. Kakyo, Xiao, and Chamberlain (2022) performed a systematic integrative review including 22 studies and found a significant difference in retention rates (Schroyer, Zellers, and Abraham 2020; Zhang et al. 2019) and intention to stay in nursing (Witter and Manley 2013) between mentored and non‐mentored nurses. In an evidence‐based literature review of 12 studies (Eckerson 2018), a high increase in nurse retention rates of 97.2% (Rosenfeld, Glassman, and Capobianco 2015), 90% (Goode, Lynn, and McElroy 2013; Medas et al. 2015; Trepanier et al. 2012), and 86% (Salmond et al. 2017), were found after the first year by using a nurse residency programme. A non‐significant increase in retention (Newhouse et al. 2007; Pillai et al. 2018) and a significant decrease in turnover (Zhang et al. 2019) were found in a systematic review (Kenny et al. 2021), by implementing Internship/externship transition programmes.

### 1.3 Rationale for the Umbrella Review

To increase the retention and decrease turnover of newly graduated nurses employed in hospital settings, knowledge is needed on the specific content of evaluated introduction programmes and the effect of the programmes. The introduction programmes are described to contain many similarities according to having specific trainers, theoretical learning and competency development of the newly graduated nurses (Edward et al. 2017; Eckerson 2018). However, heterogeneity in the content can also be present because the programmes were developed in different settings and context. Furthermore, evidence on the effect of introduction programmes for newly graduated nurses in hospital practice has been presented in numerous systematic reviews (Kakyo, Xiao, and Chamberlain 2022; Eckerson 2018; Kenny et al. 2021). However, knowledge is needed on which interventions are effective. To address the comprehensive content and evidence base of introduction programmes for newly graduated nurses in hospital settings, an umbrella review was chosen for this study to combine and compare the evidence from systematic reviews.

## 2 The Review

### 2.1 Aims

The aim of this study is to combine and compare the results from systematic reviews reporting the content and effect of introduction programmes for newly graduated nurses employed in hospital settings on increasing retention and decreasing turnover.

The research questions of the umbrella review were as follows:

- What do introduction programmes with the aim to support retention and decrease turnover contain?
- Which interventions evaluating the introduction programmes are effective in increasing retention and decreasing turnover of newly graduated nurses employed in hospital settings?

### 2.2 Design

The umbrella review followed the methodological guidance for umbrella reviews (Aromatis et al. 2015). Due to the large amount of systematic reviews conducted on the study subject, the umbrella methodology was chosen to combine and compare the existing evidence. AMSTAR 2 (A MeaSurement Tool to Assess systematic Reviews) (Shea et al. 2017) recommendations were followed for details on reporting the study (see File S1). The protocol for the umbrella review is registered with Open Science Framework (https://OSF.IO/DXYS4).

### 2.3 Search Strategy

The search strategy was performed in the databases of PubMed/MEDLINE, CINAHL and PhycInfo, which were searched in January 2023 for potentially relevant and eligible systematic reviews. According to Aromatis et al. (2015) guidance for umbrella reviews, the search should be broad to gain as many findings as possible. The search string consisted of keywords extracted from the PICOS (Population, Intervention, Comparison, Outcome, Study design) inclusion criteria (Aromatis et al. 2015) and no limiters was set on language or publication year. A PRISMA (Preferred Reporting Items for Systematic Reviews and Meta‐analysis of Studies that evaluate healthcare interventions) flowchart was used to illustrate the comprehensive search (Liberati et al. 2009).

#### 2.3.1 Inclusion and Exclusion Criteria

Criteria for inclusion of systematic reviews were as follows. Newly graduated nurses of all ages employed in all areas of hospital settings comprised the selected population. Systematic reviews reporting interventions aiming to increase retention and reduce turnover of newly graduated nurses employed in hospital settings through introduction programmes were included. The reviews were included, if the introduction programmes focused on introduction of any kind for the newly graduated nurses to clinical practice from the first day of their employment. No limits were set on the duration of the programmes. Systematic reviews reporting studies of all comparisons with the introduction programmes were included. The inclusion criteria for outcomes comprised retention and/or turnover as primary outcomes. Systematic reviews, with or without meta‐analysis, evaluating randomised controlled trials, controlled trials and experimental trials with either pre/post‐tests and/or control groups were included.

All reviews reporting non‐experimental and descriptive studies, of both qualitative and quantitative nature, were excluded.

#### 2.3.2 Search String

The search string for identification of eligible systematic reviews is presented in Table 1.

**TABLE 1:** Search string for umbrella review.

Main inclusion criteria	Search no. #	Search terms
Population	#1	Nurse OR nurses OR “registered nurses” OR “registered nurse” OR “medical nurses” OR “medical nurse”
Intervention	#2	Onboarding OR “Onboarding program” OR “Onboarding programs” OR “Onboarding process” OR “Onboarding processes” OR transition OR introduction OR mentor OR mentorship OR retention OR “nurse retention” OR staffing OR preceptor OR preceptorship OR attrition OR turnover OR “nurse turnover” OR staffing OR “nurse staffing” OR “nurse shortage”
Comparison		—
Outcomes		—
Study design	#3	“Systematic review”
	#4	#1 AND #2 AND #3
Language		English, Danish, Swedish, Norwegian
Publication year		No limiters

### 2.4 Data Extraction

To extract data from the included reviews, both authors scrutinised the reviews for data relevant to the research questions of the umbrella review.

To provide context to the results, data on (a) author(s) year, (b) included intervention studies, year limits and countries, (c) review aim, (d) population in intervention studies, (e) context, (f) introduction programmes in included review, (g) outcomes (no. studies reporting/no. studies with positive effect) and (h) study design of the review interventions were extracted from the included reviews. In extracting the study design, the numbers of included RCTs were counted as well as additional intervention designs. According to (b) the countries of the intervention studies origin were explicated.

The number of intervention studies in the included reviews was scrutinised according to overlap in studies and the number of studies that were unique to each review.

Detailed inspection of included systematic reviews was performed to identify and classify the content and effect of the unique intervention studies on retention and turnover. To source and record the content of the introduction programmes, the authors extracted data focusing on each programme reported in the reviews. Data related to the author of the review, the number of studies (and unique interventions) and the content of the programmes were afterwards reported according to the specific programmes. Data on the effect of the introduction programmes were extracted according to the main outcomes of retention and turnover, respectively. The type of intervention and study design and the positive results of the studies as well as on negative or no effects were extracted and reported. Extracting data from systematic reviews can be difficult when the methodology does not allow data inclusion from the original studies (Aromatis et al. 2015). As an example, the methods for calculating retention and turnover were not always clear in the included reviews. We therefore reported the results in percentage directly from the systematic reviews included.

### 2.5 Data Synthesis

The process and results of the umbrella review were presented through a narrative description (Aromatis et al. 2015). The narrative description relied primarily on the use of words and text to summarise and explain the findings according to the aim and research questions of the umbrella review.

### 2.6 Quality Appraisal

The AMSTAR 2 instrument (Shea et al. 2017) was used for critical appraisal of the methodological quality of the eligible systematic reviews. The AMSTAR 2 instrument includes 16 items, where a positive result is rated with “yes”, a negative with “no” and a “partial yes” response, where a partial adherence to the standard is considered (Shea et al. 2017). The instrument is not intended to combine the results in an overall score but to consider the impact of each rating. Seven items on the AMSTAR 2 (Shea et al. 2017) instrument were given specific considerations as they may critically affect the validity of a review and its conclusions. The rationale for a specific identification of the seven items is to address and assess the possible risk of bias in the included studies (Shea et al. 2017). The seven items are: Protocol registered before the commencement of the review (item 2). Adequacy of the literature search (item 4). Justification for excluding individual studies (item 7). Risk of bias from individual studies being included in the review (item 9). Appropriateness of meta‐analytical methods (item 11). Consideration of risk of bias when interpreting the results of the review (item 13). Assessment of presence and likely impact of publication bias (item 15) (Shea et al. 2017). Both authors assessed the methodological quality of the included systematic reviews. Author inter‐rater reliability was secured by both authors agreeing on how to perform the assessment (Saal, Downey, and Lahey 1980). Inter‐rater reliability was not measured for this study however, the authors compared results of their quality assessment of the included reviews and any discrepancies were resolved through discussions.

## 3 Results of the Search

### 3.1 Search Results

The comprehensive search in electronic databases of PubMed/MEDLINE, CINAHL and PhycInfo yielded 2571 systematic reviews. Titles and abstracts from all reviews were screened for eligibility and potentially relevant reviews were retrieved in full, with their citation details imported into the Covidence systematic review software (Veritas Health Innovation, Melbourne, Australia). After the screening process, 2546 reviews were excluded for not meeting the inclusion criteria, leaving 13 relevant reviews after duplicates were removed. Full‐text eligibility was assessed for the remaining 13 reviews according to the PICOS inclusion criteria. Eight reviews were excluded for not meeting the full‐text inclusion criteria of population (*n* = 1) (Lartey, Cummings, and Profetto‐McGrath 2014), outcomes (*n* = 2) (Putra, Kusnanto, and Yuwono 2020; Speight et al. 2019) and study design (*n* = 5) (Edward et al. 2017; Edwards et al. 2015; Kenny et al. 2021; Marufu et al. 2021; Van Camp and Chappy 2017).

**FIGURE 1:**
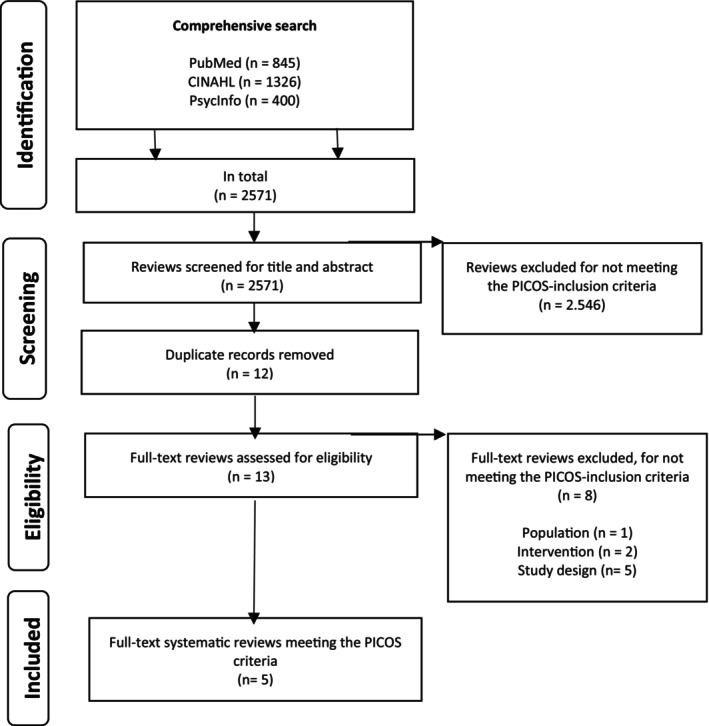
PRISMA flow of the search strategy for eligible systematic reviews.

Five full‐text reviews met the PICOS inclusion criteria. Finally, five systematic reviews were included in the umbrella review (Brook et al. 2019; Chen and Lou 2014; Ke, Kuo, and Hung 2017; Missen, Mckenna, and Beauchamp 2014; Zhang et al. 2016). Zotero (Parabhoi, Seth, and Pathy 2017) was used to manage the bibliographic records (Figure 1).

### 3.2 The Characteristics of the Included Systematic Reviews

The five included systematic reviews (Brook et al. 2019; Chen and Lou 2014; Ke, Kuo, and Hung 2017; Missen, Mckenna, and Beauchamp 2014; Zhang et al. 2016) were published from 2014 to 2019 (Table 2).

**TABLE 2:** Characteristics of included systematic reviews (*n* = 5).

Author(s) (year)	Included interventions in reviews (*N*), year limits and countries	Populations in the review interventions	Context	Introduction programmes in the review interventions (*n*)	Outcomes (retention and turnover) reported (*n*), and amount with positive or significant effect (*n*)	Study designs of the review interventions
Brook et al. (2019)	*N* = 53 (2001–2018) Australia (*n* = 2) Canada (*n* = 1) Taiwan (*n* = 1) UK (*n* = 1) USA (*n* = 48)	Newly graduated nurses and newly licensed/qualified nurses	The hospital size in which the interventions took place ranged from 12 to 1800 beds Eleven studies reported interventions in children's hospitals and 8 were multi‐site studies including a mixture of specialities	Orientation programme (*n* = 15) Residency/internship programme (*n* = 14) Mentorship programme (*n* = 7) Preceptorship programme (*n* = 4) Externship (*n* = 2) Clinical ladder advancement (*n* = 2) Other (*n* = 9)	*Retention* Reported in (*n* = 17) Positive effect in (*n* = 14) (no significant) *Turnover* Reported in (*n* = 34) Positive effect in (*n* = 26) (no significant)	RCT *n* = 1 Quasi experimental design *n* = 1 Pre‐ and post‐test *n* = 21 Post‐test only *n* = 8 Time series *n* = 22 (of these non‐equivalent control groups *n* = 7)
Chen and Lou (2014)	*N* = 5 (2001–2010) Taiwan (*n* = 3) Thailand (*n* = 1) USA (*n* = 1)	Newly registered nurses	Hospital settings (not specified)	Mentorship programme (*n* = 5)	*Turnover* Reported in (*n* = 2) Positive effect in (*n* = 2) (two significant)	Quasi‐experimental or pre‐post‐test design (*n* = 5)
Ke, Kuo, and Hung (2017)	*N* = 6 (2001–2014) China (*n* = 1) New Zealand (*n* = 1) Taiwan (*n* = 2) Thailand (*n* = 1) USA (*n* = 1)	Newly graduated nurses	Medical and surgical hospital wards (*n* = 3). Unspecified hospital settings (*n* = 3)	Preceptorship programme (*n* = 6)	*Retention* Reported in (*n* = 2) Only observed once – no results	RCT (*n* = 1) Quasi‐experimental design (*n* = 1) Observational studies (*n* = 4)
Missen, Mckenna, and Beauchamp (2014)	*N* = 11 (2001–2012) USA (*n* = 11)	Newly educated nurses	Medical hospital settings (*n* = 5) Acute hospital settings (*n* = 3) Paediatric settings (*n* = 2) Naval hospital (*n* = 1)	Internship (*n* = 2) Residency (*n* = 6) Orientation programme (*n* = 2) Integration programme (*n* = 1)	*Retention* Reported in (*n* = 3) Positive effect in (*n* = 3) (one significant) *Turnover* Reported in (*n* = 6) Positive effect in (*n* = 6) (two significant)	Quasi‐experimental design (*n* = 2) Non‐experimental, pre‐ and post‐testing (*n* = 8)
Zhang et al. (2016)	*N* = 9 (2001–2014) China (*n* = 2) Thailand (*n* = 1) USA (*n* = 6)	Newly graduated nurses	Hospital settings (not specified)	Mentorship programme (*n* = 9)	*Turnover* Reported in (*n* = 4) Positive effect in (*n* = 4) (no significant)	RCT (*n* = 1) Quasi‐experimental design (*n* = 8)

The 84 intervention studies included in the five reviews were published from 2001 to 2018 in Australia (*n* = 2), Canada (*n* = 1), China (*n* = 3), New Zealand (*n* = 1), Taiwan (*n* = 6), Thailand (*n* = 3), United Kingdom (*n* = 1) and the majority in the USA (*n* = 66). Populations in the interventions were newly employed nurses, categorised in the reviews as newly graduated nurses, newly licensed/qualified nurses and newly educated nurses. The context of the 84 interventions varied from children's hospitals, medical and surgical hospital wards, acute hospital settings, and unspecified hospital settings (Table 2). The interventions in the five reviews evaluated nine types of introduction programmes (Preceptorship programme, Mentorship programme, Residency/Internship programmes, Residency, Internship, Externship, Orientation programme, Integration programme, Clinical ladder advancement). All reviews reported outcomes of retention (*n* = 3) (Brook et al. 2019; Ke, Kuo, and Hung 2017; Missen, Mckenna, and Beauchamp 2014) and turnover (*n* = 4) (Brook et al. 2019; Chen and Lou 2014; Missen, Mckenna, and Beauchamp 2014; Zhang et al. 2016). The study designs of the 84 intervention studies included in the five reviews were randomised controlled trials (*n* = 3), quasi‐experimental trials (*n* = 17), pre and post‐test (*n* = 29), post‐test only (*n* = 8), time series (*n* = 22), observational studies (*n* = 4) and non‐experimental repeated measures design (*n* = 1) (Table 2).

### 3.3 Methodological Quality—AMSTAR

The methodological quality of the five included reviews varied but was all of low quality (Table 3).

**TABLE 3:** AMSTAR 2 critical appraisal of the quality of the included reviews (*N* = 5).

Items	Brook et al. (2019)	Chen and Lou (2014)	Ke, Kuo, and Hung (2017)	Missen, Mckenna, and Beauchamp (2014)	Zhang et al. (2016)
1. Did the research questions and inclusion criteria for the review include the components of PICO?	No	No	Yes	Yes	No
2. Did the report of the review contain an explicit statement that the review methods were established before the conduct of the review and did the report justify any significant deviations from the protocol?^a^	No (no protocol)	No (no protocol)	No (no protocol)	No (no protocol)	No (no protocol)
3. Did the review authors explain their selection of the study designs for inclusion in the review?	No	Yes	No	Yes	No
4. Did the review authors use a comprehensive literature search strategy?^a^	Partial yes	Partial yes	Partial yes	Partial yes	Partial yes
5. Did the review authors perform study selection in duplicate?	Yes	Yes	Yes	Yes	Yes
6. Did the review authors perform data extraction in duplicate?	Yes	No	No	No	Yes
7. Did the review authors provide a list of excluded studies and justify the exclusions?^a^	No	No	No	No	No
8. Did the review authors describe the included studies in adequate detail?	Partial yes	Yes	Partial yes	Partial yes	No
9. Did the review authors use a satisfactory technique for assessing the risk of bias (RoB) in individual studies that were included in the review?^a^	No	No	Yes	No	No
10. Did the review authors report on the sources of funding for the studies included in the review?	No	No	No	No	No
11. If meta‐analysis was performed did the review authors use appropriate methods for statistical combination of results?^a^	No meta‐analysis	No meta‐analysis	No meta‐analysis	No meta‐analysis	No meta‐analysis
12. If meta‐analysis was performed, did the review authors assess the potential impact of RoB in individual studies on the results of the meta‐analysis or other evidence synthesis?	No meta‐analysis	No meta‐analysis	No meta‐analysis	No meta‐analysis	No meta‐analysis
13. Did the review authors account for RoB in individual studies when interpreting/discussing the results of the review?^a^	No	No	Yes	No	No
14. Did the review authors provide a satisfactory explanation for, and discussion of, any heterogeneity observed in the results of the review?	Yes	Yes	Yes	No	No
15. If they performed quantitative synthesis did the review authors carry out an adequate investigation of publication bias (small study bias) and discuss its likely impact on the results of the review?^a^	No meta‐analysis	No meta‐analysis	No meta‐analysis	No meta‐analysis	No meta‐analysis
16. Did the review authors report any potential sources of conflict of interest, including any funding they received for conducting the review?	Yes	Yes	Yes	Yes	No

^a^ Items considered to critically affect validity.

Of a possible positive score of 16, only one review scored positive in eight items (Ke, Kuo, and Hung 2017). Two reviews scored positive in six of the AMSTAR 2 items (Brook et al. 2019; Chen and Lou 2014), one scored five (Missen, Mckenna, and Beauchamp 2014) and one scored three (Zhang et al. 2016). All reviews received a positive score for performing study selection in duplicate (item 6) and none of the reviews scored positive on reporting sources of funding for the studies included in the review (item 10).

Seven of the AMSTAR 2 items were considered potentially critical for validity (Shea et al. 2017) and were given a specific focus in the quality appraisal. Collectively, the reviews had positive scores on having a comprehensive literature search strategy (item 4). However, none of the reviews had explicit statements on the methodological establishment of protocols before the review (item 2), provided a list of excluded studies that justified the exclusions (item 7), or accounted for the risk of bias when interpreting and discussing the results of the review (item 13). Only one study (Ke, Kuo, and Hung 2017) used a satisfactory technique for assessing the risk of bias in the individual studies (item 9). No meta‐analyses were performed in the five reviews, so Items 11 and 15 were deemed irrelevant (Table 3).

Four of the five included reviews performed a quality appraisal on their included intervention studies (Brook et al. 2019; Ke, Kuo, and Hung 2017; Missen, Mckenna, and Beauchamp 2014; Zhang et al. 2016) and received low to moderate appraisal scores. The most critical bias detected were low response rate (Missen, Mckenna, and Beauchamp 2014), high attrition rate (Missen, Mckenna, and Beauchamp 2014), absence of information (Brook et al. 2019), detection bias (Brook et al. 2019), unclear methods used for data collection (Missen, Mckenna, and Beauchamp 2014), lack of random assignment (Ke, Kuo, and Hung 2017), lack of concealed allocation (Ke, Kuo, and Hung 2017) and lack of blinding of participants and outcome assessors (Ke, Kuo, and Hung 2017; Zhang et al. 2016) (Table 3).

### 3.4 Overlap Among Reviews

A total of 84 intervention studies were included in the five reviews (Brook et al. 2019; Chen and Lou 2014; Ke, Kuo, and Hung 2017; Missen, Mckenna, and Beauchamp 2014; Zhang et al. 2016). Among the 84 intervention studies 14 studies (16.7%) overlapped in the reviews and 70 studies were unique. However, three of the overlapping studies (Latham, Ringl, and Hogan 2011; Lin and Tsai 2010; Newhouse et al. 2007) were not problematic as Lin and Tsai (2010) did not report retention or turnover, Latham, Ringl, and Hogan' (2011) results on retention was only reported in one review (Brook et al. 2019), and Newhouse et al. (2007) were reported for retention (Brook et al. 2019) and turnover (Missen, Mckenna, and Beauchamp 2014) respectively. Recommendations considering data extracted from different sources for the same primary study (Lunny et al. 2021) were taken into account during the data synthesis by avoiding the double presentation of intervention studies.

## 4 Results of the Data Synthesis

### 4.1 Content of the Introduction Programmes

The content of the nine introduction programmes in the five systematic reviews is presented in Table 4.

**TABLE 4:** The content of the programmes for introduction.

Programmes for introduction	Author(s) (year)	No. of studies/duration	Content of the programmes
Preceptorship programme	Brook et al. (2019)	*N* = 4	The newly qualified nurse is allocated a qualified nurse preceptor and regular meetings are arranged
			Involve formal teaching, supervision and competency assessment
		From 8 weeks to 1 year	Relationship between preceptor and preceptee is fundamental for psychological support, knowledge exchange and role modelling
	Ke, Kuo, and Hung (2017)	*N* = 6	Fixed preceptors/preceptee one‐on‐one
		From 1 month to 2 years	Preceptor training workshops on: Introduction to and understanding of the roles, responsibilities and function of preceptors and the preceptorship programme Professional socialisation and reality shock Principles of adult learning, motivation and strategies Conflict‐ and stress management Problem‐solving and decision‐making Hospital policies Common nursing process
Mentorship programme	Brook et al. (2019)	*N* = 7	Include dyad models, peer mentoring, group mentoring, constellation models or online distance mentoring
		From 4 weeks to 1 year	Mentors may be allocated to mentees or may rely on self‐selection or incentives to motivate tenacity in the relationship
	Chen and Lou (2014)	*N* = 5	Newly graduated nurses had one regular mentor in all studies, who had completed training before the intervention on:
		From 3 months to 1 year	Establishing a mentorship relationship Asking newly registered nurses to observe, respond, demonstrate their skills, and practice and prepare for lessons before class Administering examinations Implementing multidimensional teaching strategies including discussions, provision of feedback, and reflection Developing interpersonal relationships and problem‐solving skills
	Zhang et al. (2016)	*N* = 9	*Mentor selection*: Criteria for mentor selection were considered crucial for success, including experienced staff nurses with at least 3 years experience, good decision‐making competencies and communication skills
		From 1 month to 3 years	*Mentor training*: On mentorship roles, occupational socialisation, reality shock, adult learning, conflict resolution, stress management, and clinical nursing skills
			*Mentor and mentee matching*: Matching emphasised that mentor and mentee should have a non‐evaluative relationship, and have similar clinical backgrounds. The mentor and mentee should possess a sense of mutual attraction as well as common interests and values
		
*Mentor and mentee relationship*: Monthly meetings between the mentor and mentee and informal meetings with the program coordinator for ongoing support to provide regular feedback and to increase the success of the mentees' transitions
Residency/internship programme	Brook et al. (2019)	*N* = 14	Contractual arrangement between the new graduate nurse and the employer
		1 year on average	Delivered in partnership between academia and practice
			Involve structured teaching linked to clinical immersion and a mentoring relationship
Residency	Missen, Mckenna, and Beauchamp (2014)	*N* = 6	Preceptor‐guided and clinical experience
			Integrating human patient simulation
		From 12 weeks to 1 year	Providing pathways that foster clinical comfort and the development of confidence and competence
			Social and professional reality integration
Internship	Missen, Mckenna, and Beauchamp (2014)	*N* = 2	All were receptor‐guided
			Clinical experience
		From 16 weeks to 1 year	Fostering clinical comfort and the development of confidence and competence
Externship	Brook et al. (2019)	*N* = 2	Opportunity for students to apply theoretical knowledge, experience the clinical environment and understand workplace opportunities
		From 10 weeks to 1 year	Focus on theory rather than practical placements
Orientation programme	Brook et al. (2019)	*N* = 15	Involve formal teaching and a mentor or preceptor relationship
		From 6 weeks to 1 year	
	Missen, Mckenna, and Beauchamp (2014)	*N* = 2	Preceptor guided
		From 6–10 weeks to 12–24 weeks	
Integration programme	Missen, Mckenna, and Beauchamp (2014)	*N* = 1	Preceptor guided with scheduled education seminars
		1 year	
Clinical ladder advancement	Brook et al. (2019)	*N* = 2	Offer clarity about professional development and promotion
			Involves competency assessment
		Variating	Usually involves remuneration for achievement

#### 4.1.1 Types of Introduction Programmes

The five reviews all presented intervention studies evaluating introduction programmes of newly graduated nurses in hospital settings (Chen and Lou 2014; Brook et al. 2019; Ke, Kuo, and Hung 2017; Missen, Mckenna, and Beauchamp 2014; Zhang et al. 2016) (Table 4). The reviews reported nine types of programmes: Preceptorship programme (Brook et al. 2019; Ke, Kuo, and Hung 2017), Mentorship programme (Brook et al. 2019; Chen and Lou 2014; Zhang et al. 2016), Residency/Internship programme (Brook et al. 2019), Residency (Missen, Mckenna, and Beauchamp 2014), Internship (Missen, Mckenna, and Beauchamp 2014), Externship (Brook et al. 2019), Orientation programme (Brook et al. 2019; Missen, Mckenna, and Beauchamp 2014), Integration programme (Missen, Mckenna, and Beauchamp 2014) and Clinical ladder advancement (Brook et al. 2019). Eight of the nine programmes were executed by a nurse in a preceptor role except the mentorship programmes, which were guided by a nurse in a mentor role. This meant that all the introduction programmes reported had either a preceptor or mentor as a trainer for the newly graduated nurses. The five included systematic reviews described a lack of reporting in the initial studies of the definition of the role as a preceptors/mentors and the criteria for selection. Only Chen and Lou (2014) reported that seniority was seen as a criterion for being a mentor. None of the included reviews reported the characteristic of the role as preceptor/mentor, apart from their training to be a preceptor/mentor, the importance of establishing a relationship with the newly graduated nurse, and the introduction elements they were providing.

The duration of the programmes lasted from 4 weeks to 3 years, however, the majority of reviews reported programmes that lasted for 1 year (Table 4).

The content of the nine programmes was directed towards training elements for the preceptor/mentor role and introduction elements for the newly graduated nurse to clinical practice. One review (Brook et al. 2019) did not describe the content of the included intervention studies but used overall definitions of the programme designs.

#### 4.1.2 Training of Preceptors/Mentors

Four reviews (Ke, Kuo, and Hung 2017; Zhang et al. 2016; Chen and Lou 2014; Missen, Mckenna, and Beauchamp 2014) described the preceptor/mentor training as a part of the introduction programmes of newly graduated nurses. The preceptors/mentors were trained to prepare the newly graduated nurses for clinical practice and to teach them about the nursing process (Ke, Kuo, and Hung 2017), to ask the newly graduated nurses to observe, respond, and demonstrate their skills (Chen and Lou 2014), and to participate in multidisciplinary patient care rounds (Missen, Mckenna, and Beauchamp 2014). Academic preparations for implementing multidimensional teaching strategies including discussions, provision of feedback, and reflections (Chen and Lou 2014) were applied to teach the newly graduated nurses to prepare for lessons before class (Chen and Lou 2014) and to be involved in of didactic presentations, simulated experiences, seminars and case study presentations (Missen, Mckenna, and Beauchamp 2014). The preceptors/mentors were also trained on the principles of adult learning and motivation (Ke, Kuo, and Hung 2017; Zhang et al. 2016). Furthermore, the training consisted of strategies for professional (Ke, Kuo, and Hung 2017) and occupational socialisation (Zhang et al. 2016) as well as being prepared to take care of reality shock, problem‐solving, conflict resolution and stress management (Ke, Kuo, and Hung 2017; Zhang et al. 2016; Chen and Lou 2014). The training programmes for the preceptors/mentors were reported to last from 2 to 16 h.

#### 4.1.3 Establishing a Relationship

The training of preceptors/mentors on how to establish a relationship with the newly graduated nurses was emphasised in all five reviews (Ke, Kuo, and Hung 2017; Zhang et al. 2016; Chen and Lou 2014; Missen, Mckenna, and Beauchamp 2014; Brook et al. 2019) as an important element in making the programme successful. The preceptors/mentors were provided with an introduction to understanding their roles, responsibilities and functions (Ke, Kuo, and Hung 2017) as well as how to establish a mentorship relationship (Chen and Lou 2014). The reviews described how the relationship between preceptor/mentor and the newly graduated nurse was fundamental for psychological support, knowledge exchange and role modelling (Brook et al. 2019). Preceptors/mentors were also found to have a strong influence on newly graduated nurses during the initial socialisation period concerning role modelling and professional behaviours (Missen, Mckenna, and Beauchamp 2014). Only one review (Zhang et al. 2016) reported how the criteria for mentor selection should be experienced staff nurses with at least 3 years of experience with good communication skills and competencies. The reviews reported different ways of matching the preceptor/mentor with the newly graduated nurse. Two reviews (Zhang et al. 2016; Brook et al. 2019) reported how the matching with mentors should be based on mutual attraction and common values (Zhang et al. 2016) and how matching the mentor and newly graduated nurse should rely on self‐selection to motivate for perseverance in the relationship (Brook et al. 2019). Three reviews (Brook et al. 2019; Ke, Kuo, and Hung 2017; Chen and Lou 2014) reported how preceptors were pre‐selected by the management.

#### 4.1.4 Introduction Elements for the Newly Graduated Nurses

Three reviews (Brook et al. 2019; Missen, Mckenna, and Beauchamp 2014; Zhang et al. 2016) reported the content of introduction elements directed towards the newly graduated nurses for their new position in the hospital setting. The elements consisted of a focus on theory through scheduled education seminars (Missen, Mckenna, and Beauchamp 2014) and structured and formal teaching and supervision (Brook et al. 2019) to provide the newly graduated nurse with the opportunity to apply theoretical knowledge to clinical immersion (Brook et al. 2019). The introduction elements also included preparation for clinical practice through social‐ and professional reality integration (Missen, Mckenna, and Beauchamp 2014), understanding the clinical environment and the workplace opportunities (Brook et al. 2019), and knowledge of pathways that foster clinical comfort (Missen, Mckenna, and Beauchamp 2014). Offering development and assessment of confidence and competencies (Brook et al. 2019; Missen, Mckenna, and Beauchamp 2014) as well as clarity about professional development and promotion (Brook et al. 2019) was also described as introduction elements for the newly graduated nurses. Zhang et al. (2016) reported the importance of having informal meetings with the program coordinator for ongoing support to provide regular feedback and to increase the success of the newly graduated nurses' transitions as well as monthly meetings with the preceptor/mentor.

### 4.2 Effects of the Introduction Programmes on Retention and Turnover

The effect of the introduction programmes on retention and turnover are presented in Table 5. The 11 overlapping intervention studies within the five systematic reviews have been considered and only the unique studies have been reported.

**TABLE 5:** Effect of programmes for introduction on retention and turnover.

Review(s)	Type of intervention and study design	Positive results of the studies	Negative or no effect
*Retention*			
Brook et al. (2019)	**Preceptorship programme (*n* = 1)**		
	Pre‐post‐test design (*n* = 1)	Overall hospital retention increased by 29% from pre (April 2000) to post (May 2001) intervention; newly graduated nurse retention increased by 68%	—
	**Mentorship programme (*n* = 2)**		
	Pre‐post‐test design (*n* = 2)	21% increase in retention for Hospital 1 pre‐intervention to 3–4 years after; 17.07% increase in retention for Hospital 2 pre‐intervention to 3–4 years after	—
		13% increase in retention 6 months post‐intervention compared to pre‐intervention	—
	**Residency/internship programme (*n* = 4)**		
	Post‐test only (*n* = 3)	40% increase in retention from pre‐intervention to post (2 years after intervention)	—
		8% improvement in retention for the intervention group versus the comparison group at 18 months post‐intervention; 12% improvement in retention at 24 months	—
		21% improvement in retention from 2 years pre‐intervention to post‐intervention	—
	Pre‐post‐test design (*n* = 1)	25% improvement in retention pre (2001) to post (2006) intervention and 11% improvement in retention from pre (2001) to post (2007) intervention	—
	**Externship (*n* = 1)**		
	Time series (*n* = 1)	—	4% decrease in retention at 1 year for externship; 14% decrease at 2 years
	**Orientation programme (*n* = 7)**		
	Time series (*n* = 5)	6% increase in retention in interventions with high levels of preceptor support compared to interventions with low levels of preceptor support	—
	
		12% improvement in retention across all paediatric nurses pre and post‐intervention	—
		20% increase in retention in the intervention group (critical care orientation) versus the control group (standard orientation)	—
		22.5% increase in retention pre (1 year before intervention) to post (5 years after adoption of intervention)	—
	1.9% increase in retention from pre (May 2005) to post‐intervention (August 2006)	—
	Post‐test only (*n* = 2)	Improvement of 20% from pre (previous 2‐year retention) to post (current 2‐year retention) intervention	—
Short‐term retention (12 months) increased by 37% for the intervention group versus the control group; long‐term retention (nurses who stayed in emergency nursing, regardless of location) increased by 49.7%		—
Ke, Kuo, and Hung (2017)	**Preceptorship programme (*n* = 2)**		
	Observational study (*n* = 2)	1st year = 78% (28/36)	—
		2nd year = 88.46% (69/78)	—
Missen, Mckenna, and Beauchamp (2014)	**Residency (*n* = 3)**		
	Pre‐post‐test design (*n* = 2)	This program has retained 87% (275 of 316) of its residents at the end of the 1‐year program	—
		The first‐year cohort's employment retention rate was 78%, and the second‐year cohort is presently 96%^a^	—
	**Integration programme (*n* = 1)**		
	Quasi experimental design (*n* = 1)	Significant difference (*p* = 0.014) between intervention‐ and control group after 12 months	—
*Turnover*			
Brook et al. (2019)	**Preceptorship programme (*n* = 4)**		
	Time series (*n* = 2)	3.9% reduction in turnover from pre (2001) to post (2004) intervention	—
	
	9% decrease in turnover at 12 months post‐intervention; 4% at 24 months post‐intervention	—
	Post‐test only (*n* = 1)	9.5% improvement between turnover in the intervention group and cohort 1; 23.1% improvement between the intervention group and cohort 2	—
	Pre‐post‐test design (*n* = 1)	Reduction in turnover of 17.7% from pre‐intervention (March 2006) to during the 6‐month study period (August 2006)	—
	**Mentorship programme (*n* = 2)**		
	Time series (*n* = 1)	15.4% decrease in turnover after one year, 18.23% decrease after two years, and 10.7% decrease in turnover three years post‐intervention	—
	Post‐test only (*n* = 1)	The average of the four participating hospitals showed that 23% of non‐participants had left within a year of hire, while only 8% of participants had left	—
	**Externship (*n* = 1)**		
Time series (*n* = 1)	—	8.8% increase in turnover for the intervention group; of the 193 nurses who completed the externship, 153 accepted a graduate nurse position at the institution
Brook et al. (2019)	**Residency/internship programme (*n* = 10)**		
	Pre‐post‐test design (*n* = 5)	8% decrease in turnover pre (2001–2002) to post (2003–2005) intervention	—
		37% decrease in turnover from pre (2003) to post (2005) intervention	2% increased turnover for intervention site at 1‐year post‐intervention compared to control sites
		Data averaged across 15 hospitals: 29.63% mean decrease in turnover from pre (12 months) to post (12 months) intervention	—
Brook et al. (2019)
	Time series (*n* = 4)	For intervention group: Mean change in turnover = decrease of 9.3%; for non‐intervention group: Mean change in turnover = increase of 14.5%	—
		7.8% decrease in turnover at 1 year post‐intervention and 9.3% at 2 years post‐intervention	—
		17.2% decrease in turnover pre (2002) to post (12 months after) intervention	—
		5% decrease in turnover from 1 year pre to 1 year post‐intervention	—
		8% decrease in turnover 2 years pre to 1 year post‐intervention; 4% decrease in turnover 2 years pre to 2 years post‐intervention	—
		19.9% decrease in turnover from pre‐intervention compared to post (12 months post‐recruitment)	—
	Post‐test only (*n* = 1)	22% reduction in turnover compared to the control group, measured over 12 month period	—
	**Orientation programme (*n* = 9)**		
	Pre‐post‐test design (*n* = 4)	13% decrease in turnover pre (July 2003–June 2004) to post (October 2004—July 2005) intervention	—
		11% decrease in turnover from pre to post (18 months after intervention)	—
		50% reduction in turnover from pre (2001/2002) to post (2002/2004) intervention	—
		14.9% reduction in turnover for the intervention group compared to the comparison group (whole hospital)	—
	Time series (*n* = 3)	2.5% decrease in turnover from pre (May 2005) to post‐intervention (August 2006)	—
		28% decrease in turnover from pre (1998) to post (2003) intervention	—
10.7% decrease in turnover from pre (2002) to post (2005) intervention		—

	RCT (*n* = 1)	Intervention group turnover rate 15.5%, control group turnover rate 26.8%	—
Quasi‐experimental design (*n* = 1)	31.1% decrease in turnover from pre (2007) to post (2009) intervention	—
Brook et al. (2019)	**Clinical ladder advancement (*n* = 2)**		
	Time series (*n* = 1)	Turnover (2006) was 9.4% less for the intervention group	—
	Pre‐post‐test design (*n* = 1)	10.8% decrease in turnover pre‐intervention to post‐intervention; turnover was 8.9% less for those on the clinical ladder programme	—
Chen and Lou (2014)	**Mentorship programmes (*n* = 2)**		
	Quasi‐experimental Pre‐post‐test design (*n* = 2)	Significant decrease (*n* = 2) not specified^b^	—
		Lower turnover in the intervention group (12%) compared to the control group (20%)^c^	—
Missen, Mckenna, and Beauchamp (2014)	**Residency (*n* = 3)**		
	Non‐experimental, repeated measures design (*n* = 1)	New nurse turnover was 7%–11% compared to previously 12%–15%^d^	—
	Pre‐post‐test design (*n* = 2)	Significant predictor of employment status (*p* ≤ 0.0001). Turnover rate 7.1% at 12 months with a further decrease of 4.3% after 10 years^e^	—
		Turnover rate decrease of 12%	—
	**Internship (*n* = 2)**		
	Pre‐post‐test design (*n* = 2)	Significant difference to control group (*p* = 0.01) after 6 months^f^	No significant difference after 12 months (*p* = 0.20)
		Lower turnover rate in the pilot group (12%) compared to (20%) in CG^g^	—
	**Integration programme (*n* = 1)**		
	Quasi‐experimental design (=1)	—	Lower anticipated turnover at 6 months and turnover rate was not significant at 12 months (*p* = 0.20)
Zhang et al. (2016)	**Mentorship programme (*n* = 4)**		

	Quasi‐experimental design (*n* = 4)	Turnover rate reduction from 20% in 2003–2004 to 7% after mentoring in 2004–2005^h^	—
		Attrition rate for nonparticipants was 23% compared to 8% for participants^i^	—
		Turnover history from 2002 to 2005 that experienced a 2% reduction after the implementation of group mentoring compared to the preceding 3 years^j^	—
Mentoring programme contributed to an 12% reduction in the turnover rate compared to the pre‐mentoring group, in which the turnover rate was 20%^k^		—

*Note:* Overlapping studies with: ^a^Ke, Kuo, and Hung (2017); ^b^Brook et al. (2019); ^c^Brook et al. (2019), Missen, Mckenna, and Beauchamp (2014), and Zhang et al. (2016); ^d^Brook et al. (2019); ^e^Brook et al. (2019); ^f^Brook et al. (2019) and Zhang et al. (2016); ^g^Brook et al. (2019), Chen and Lou (2014), and Zhang et al. (2016); ^h^Brook et al. (2019); ^i^Brook et al. (2019); ^j^Brook et al. (2019); ^k^Brook et al. (2019).

#### 4.2.1 Retention

Three reviews (Brook et al. 2019; Missen, Mckenna, and Beauchamp 2014; Ke, Kuo, and Hung 2017) reported 20 (19 unique) intervention studies evaluating introduction programmes with retention as a primary outcome. Eighteen of 19 intervention studies found an increase in the retention of newly graduated nurses (Brook et al. 2019; Missen, Mckenna, and Beauchamp 2014; Ke, Kuo, and Hung 2017). The range of increase in retention was reported in the three reviews to be after 6 months (13.0%), 1 year (1.9%–87.0%), 18 months (8%), 2 years (12.0%–88.46%), 3 years (17%–21%), 5 years (22.5%–25.0%) and after 6 years (11%) (Brook et al. 2019; Missen, Mckenna, and Beauchamp 2014; Ke, Kuo, and Hung 2017). One review (Missen, Mckenna, and Beauchamp 2014) reported significant differences (*p* = 0.014) between the intervention and control group after 1 year based on a quasi‐experimental design. The highest increase in retention of newly graduated nurses was reported from two Preceptorship programmes, with a mean increase of 78% after 1 year and 88% after 2 years, respectively (Ke, Kuo, and Hung 2017) and from a Residency programme retaining 87% (275 of 316) of the newly graduated nurses at the end of the 1‐year program (Missen, Mckenna, and Beauchamp 2014). Only one of the intervention studies found negative results after evaluating an Externship model and found a decrease in retention of newly graduated nurses of 4% after 1 year and 14% after 2 years (Brook et al. 2019).

#### 4.2.2 Turnover

Four reviews (Brook et al. 2019; Chen and Lou 2014; Missen, Mckenna, and Beauchamp 2014; Zhang et al. 2016) reported 40 (29 unique) intervention studies evaluating introduction programmes with turnover as the primary outcome. Twenty‐seven unique intervention studies were reported in the reviews with large variations in their decrease in turnover. The range of decrease in turnover was after 6 months (17.7%), 1 year (2.5%–29.36%), 18 months (11%), 2 years (4.0%–37.0%), 3 years (3.9%–50.0%), 4 years (8%) and after 5 years (28%) (Brook et al. 2019; Chen and Lou 2014; Missen, Mckenna, and Beauchamp 2014; Zhang et al. 2016). One review reported interventions with a significant decrease in turnover between groups after 6 months for an Internship programme (*p* = 0.01) and of 7.1% after 1 year for a Residency programme (*p* = < 0.0001) (Missen, Mckenna, and Beauchamp 2014). The highest decrease in turnover was found from Residency programmes and internships combined with an average decrease in turnover of 17% after 1 year (Brook et al. 2019) and from orientation programmes with a mean decrease in turnover of 41% after 2 years (Brook et al. 2019). Two reviews (Brook et al. 2019; Missen, Mckenna, and Beauchamp 2014) reported negative results from four unique intervention studies showing an increase in nursing turnover. Brook et al. (2019) reported an 8.8% increase in turnover for the intervention group in one study, as only 153 of 193 newly graduated nurses accepted a position after their externship, and a 2% increased turnover for the intervention site at 1 year post‐intervention compared to control sites in another study. Missen, Mckenna, and Beauchamp (2014) reported a study showing no significant difference after 12 months (*p* = 0.20) after an Internship programme and a second study where the turnover rate was not significant at 12 months (*p* = 0.20).

## 5 Discussion

The majority of interventions evaluated Mentorship programmes and Preceptorship programmes for the introduction. The Preceptorship and Mentorship programmes presented heterogeneity in content related to extensive training of preceptors/mentors, and formal teaching and supervision of the newly graduated nurses (Brook et al. 2019; Ke, Kuo, and Hung 2017; Zhang et al. 2016). Differences appeared however in the selection of the preceptor/mentor as well as the content of the roles (Brook et al. 2019; Ke, Kuo, and Hung 2017; Zhang et al. 2016). Preceptors were often pre‐selected by the department management before the newly graduated nurse was enrolled in the Preceptorship programme (Brook et al. 2019; Ke, Kuo, and Hung 2017). This is supported by the literature that further describes how the commitment to preceptorship is short‐term and often ends after 6 weeks (Wensel 2006). The relationship between the preceptor and the preceptee is often strictly professionally prearranged by the organisation (Wensel 2006). In the Mentorship programmes, mentors are predominantly selected by the newly graduated nurses (Brook et al. 2019; Zhang et al. 2016). The relationship between a mentor and mentee is “naturally formed”, and designed to promote professional and personal development, provide direction and foster self‐confidence (Wensel 2006). The Mentorship programmes were reported to hold a specific focus on introduction to practice as well as the social interaction with their new workplace (Brook et al. 2019; Zhang et al. 2016), including the importance of establishing a close relationship between the mentor and the mentee. This is supported by research describing how social interaction (Bell and Sheridan 2020) and social connection and place integration (Cosgrave, Malatzky, and Gillespie 2019) are essential in the retention of nurses. This could be the reason why Mentorship programmes were more successful in achieving effective outcomes than preceptorships. The introduction programmes also focused on the newly graduated nurses' transition to clinical practice since providing direct care in a hospital setting can be a stressful and uncertain process for them as they need support in doing so (Kaldal et al. 2023). Training in understanding the clinical environment (Brook et al. 2019) and professional reality integration (Missen, Mckenna, and Beauchamp 2014) was implemented to aspire confidence for the newly graduated nurses as well as to increase their clinical competencies in patient care (Brook et al. 2019; Missen, Mckenna, and Beauchamp 2014).

A mean average of increased retention was found in 19 intervention studies (Brook et al. 2019; Missen, Mckenna, and Beauchamp 2014; Ke, Kuo, and Hung 2017) and 38 intervention studies found significant mean variations in decreased turnover (Brook et al. 2019; Chen and Lou 2014; Missen, Mckenna, and Beauchamp 2014; Zhang et al. 2016). The large effect variation could be caused by the different approaches and content within the introduction programmes. The specific content of the programmes reported in the five reviews was sparsely described as overall definitions. We can therefore not conclude that a specific programme is effective in increasing retention and reducing turnover of newly graduated nurses. However, the single content elements of the introduction programmes could have an important effect on retention and turnover of newly graduated nurses such as teaching and training for the preceptors/mentors and a solid relationship between the preceptor/mentor and newly graduated nurse. A systematic review by Marufu et al. (2021) reported common factors that influence the retention of nurses, such as education and career advancement aspects, support at work, and personal factors. These factors were covered in the majority of introduction programmes through the preceptors/mentors focus on the newly graduated nurses. However, the programmes did not cover the influence of factors such as nursing management, staffing issues, work environment issues, and financial aspects (Marufu et al. 2021) on newly graduated nurses' retention. These factors could be considered as confounders to the variations in increased retention. Research shows that personal relations with colleagues and managers are a special indicator of nurses' intentions to stay (Heidari, Seifi, and Gharebagh 2017). This was also found in a qualitative interview study of 22 nurses in a University Hospital Medical department in Denmark (Berthelsen et al. 2024). The findings showed that three of the nurses' five essential factors to stay were based on personal relations with colleagues or family, flexible working hours and work‐life balance, and feeling of being in a collegial unity (Berthelsen et al. 2024). Essentially, work environment, culture and local context are important factors that need to be considered additionally to introduction programmes.

Strategies for retention outlined in our umbrella review point to the necessity of comprehensive introduction programmes with teaching, proper preparation and training for the preceptors/mentors and a solid relationship between the preceptor/mentor and newly graduated nurse as core elements. Although the reviews report increased retention (Ke, Kuo, and Hung 2017; Brook et al. 2019; Missen, Mckenna, and Beauchamp 2014) and decreased turnover (Zhang et al. 2016; Chen and Lou 2014) by implementing introduction programmes of various kinds, we cannot say with certainty that this improvement is caused by specific elements, or the programmes alone and not by related confounders, as there are uncertainties about association between content and effect. However, tendencies can show how introduction to newly graduated nurses may influence retention and turnover when it includes creating a supportive work culture, providing professional development opportunities, and addressing issues related to workload and work‐life balance (Marufu et al. 2021). To increase retention and decrease turnover, we need stronger intervention designs to evaluate specific introduction programmes with precisely described content and process elements and duration of the elements. Additional considerations such as organisational settings, local context and culture, management and work environmental factors must be made, to avoid confounding influences.

### 5.1 Strength and Limitations

To the best of our knowledge, this is the first umbrella review conducted to compare and contrast results from systematic reviews reporting the content and effect of interventions aiming to increase retention and decrease turnover of newly graduated nurses employed in hospital settings.

The strength of the umbrella review is the inclusion of 84 intervention studies within five included systematic reviews due to the vast amount of evaluations of introduction programmes. A limitation of the umbrella design can be produced if an overlap of primary studies within the included reviews is not considered and leads to results being presented multiple times (Lunny et al. 2021). For this umbrella, 14 primary intervention studies were identified as overlapping within the included reviews. The data synthesis was therefore performed and presented on the 70 unique intervention studies.

The five reviews had a general lack of clarity on reporting the duration of the single programme elements, the order of performance of elements, and learning objectives for the newly graduated nurses. The lack of clarity presented a limitation to performing a thorough interpretation of the review results due to the limited reporting of the original studies in the reviews. Other reviews on the subject conclude, that clear conclusions regarding the effect of programmes on newly graduated nurses' retention rate could not be made because of inconsistent time points and a lack of control groups in the study design (Ke, Kuo, and Hung 2017), and how a lack of consistently reported studies limits definitive conclusions (Kenny et al. 2021). The lack of clarity of the reported interventions could be associated with the low quality of the intervention design. The majority of included intervention studies in the five reviews were quasi‐experimental designs without blinding or randomisation which could affect the accuracy of the results inference with biased estimates of effects (Altman et al. 2001) and be a threat to generalisability to other groups or hospital settings (Polit and Beck 2018). Furthermore, the five reviews were all evaluated to be of low quality according to the AMSTAR 2 assessment (Shea et al. 2017). The consequences of the low‐quality rating of the included reviews could be a threat to the validity of the results in this study.

Due to the included reviews' lack of clarity in reporting and the bias of the intervention studies, it can be difficult to conclude which precise introduction programmes were effective in increasing retention and decreasing turnover of newly graduated nurses employed in hospital settings.

## 6 Conclusion

Nine types of introduction programmes were evaluated in the five included systematic reviews of this umbrella review. Mentorship programmes and Preceptorship programmes were the most frequently utilised and evaluated programmes in the included intervention studies of the five reviews. Although the nine introduction programmes showed overall positive effects on retention and turnover, the lack of transparency of the reviews in reporting the content of the introduction programmes as well as the bias of the intervention studies within the reviews, made it difficult to conclude what content of the specific programmes used were effective. However, a close relationship between the mentor and the newly graduated nurse and the social connection was seen as an essential factor for retention and could be the reason why the Mentorship programmes seemed more effective than other introduction programmes. It could also, cautiously, be concluded that introduction programmes focusing on teaching, training for the preceptors/mentors, teaching of the newly graduated nurses and a solid relationship between the two, can have a positive impact on the retention and turnover of newly graduated nurses employed in hospital settings.

### 6.1 Implications for Practice, Policies and Research

The results of this umbrella review have several implications for practice and policies. The retention and turnover of nurses is a crucial aspect of healthcare practice, and it has several implications for both clinical practice and the healthcare system as a whole (Efendi et al. 2019). Nurse retention is essential for maintaining high‐quality patient care (Cho et al. 2020), controlling costs (Eckerson 2018), fostering a positive work environment (Marufu et al. 2021), and ensuring the overall stability and effectiveness of healthcare organisations (Drennan and Ross 2019). However, further research is needed to establish knowledge on which specific introductory elements are important to retain newly graduated nurses in their positions.

## Author Contributions

**Connie Berthelsen:** conceptualization, formal analysis, investigation, project administration, supervision, validation, visualisation, writing – original draft, writing – review & editing. **Carrinna Aviaja Hansen:** conceptualization, validation, visualisation, writing – review & editing.

## Conflicts of Interest

The authors declare no conflicts of interest.

## Supporting information


**File S1.** AMSTAR 2_Reporting

## Data Availability

The authors have nothing to report.
